# Antidiabetic and Antiobesity Effects of Artemether in db/db Mice

**DOI:** 10.1155/2018/8639523

**Published:** 2018-05-13

**Authors:** Yu Guo, Wei Fu, Yakai Xin, Jinlei Bai, Huifang Peng, Liujun Fu, Jie Liu, Liping Li, Yujin Ma, Hongwei Jiang

**Affiliations:** ^1^Department of Endocrinology, The First Affiliated Hospital and College of Clinical Medicine of Henan University of Science and Technology, Luoyang 471003, China; ^2^Key Laboratory of Endocrinology Inherited and Metabolic Diseases of Luoyang, Luoyang 471003, China; ^3^Clinical Medicine Research Center of Endocrine and Metabolic Disease of Luoyang, Luoyang 471003, China; ^4^Academican Workstation for Diabetic Kidney Disease Research of Henan Province, Luoyang 471003, China

## Abstract

This study is designed to investigate the effect of artemether on type 2 diabetic db/db mice. The experiments consisted of three groups: normal control (NC, db/+, 1% methylcellulose, intragastric administration), diabetic control (DM, db/db, 1% methylcellulose, intragastric administration), and artemether treated (artemether, db/db, 200 mg/kg of artemether, intragastric administration). The treatment lasted for two weeks. The food intake, body weight, and fasting blood glucose of mice were measured every three days. At the start and end of the experiment, the intraperitoneal glucose tolerance test (IPGTT) and insulin tolerance test (IPITT) were performed. We determined the serum insulin and glucagon levels by ELISA kits and calculated insulin resistance index (HOME-IR). HE staining was used to observe the morphologies of pancreas and liver in mice. The damage of pancreatic beta cells was evaluated by TUNEL staining and immunofluorescence. We found the following: (1) compared with the DM group, the food intake and weight increase rate of artemether group significantly reduced (*P* < 0.05); (2) compared with pretreatment, artemether significantly reduced the fasting blood glucose levels, and the areas under the curves (AUCs) of IPGTT were decreased significantly, increasing the tolerance to glucose of db/db mice. (*P* < 0.05); (3) artemether improved hyperinsulinemia and decreased the AUCs of IPITT and HOME-IR, increasing the insulin sensitivity of db/db mice. (4) Artemether significantly ameliorated islet vacuolar degeneration and hepatic steatosis in db/db mice. (5) Artemether reduced the apoptosis of pancreatic beta cells and increased insulin secretion in db/db mice compared with DM group (*P* < 0.05). Our results indicated that artemether significantly improved glucose homeostasis and insulin resistance and had the potential activity to prevent obesity, reduced the severity of fatty liver, and protected pancreatic beta cells, promising to treat type 2 diabetes.

## 1. Introduction

Diabetes mellitus is one of the most refractory metabolic disorders characterized by increased blood glucose level, as a result of an absolute or relative lack of insulin and failure of insulin to act on targets tissue [1]. In general, the majority of diabetes mellitus can be divided into type 1 and type 2 diabetes. They have different pathogeneses. The main pathogeneses of type 2 diabetes mellitus are failure of insulin secretion and insulin resistance [2]. Long-term hyperglycemia leads to pancreatic beta cells apoptosis and insulin resistance [3]. At present, some medicines such as sulfonylurea, biguanides, thiazolidinediones, and glycosidase inhibitors are widely used in clinic to control the symptoms of hyperglycemia and insulin resistance in patients with type 2 diabetes. However, the efficacy of these drugs in the treatment of type 2 diabetes is limited and they may have some side effects, such as increasing the appetite and weight [4]. In recent years, some Chinese herbs have been found to treat diabetes.

Artemisinin is obtained from the Chinese medicinal herb* Artemisia annua*, for its potent antimalarial activity [5]. Apart from the antimalarial property, the therapeutic applications of artemisinin and its derivatives (including artesunate, artemether, artenimol, and artelinic acid) are wide and huge [6]. Artemisinins have also been found to be associated with many other activities including anticancer activity, anti-inflammatory activity, and antibacterial and antiviral activity [7–9]. However, the effect of artemisinins on diabetes is largely unknown.

Previous animal studies have found that artemisinins possessed hypoglycemic, antihyperlipidemic effects in STZ induced diabetic mice, and even valuable effects on liver and renal functions [10, 11]. Artemisia extract also alleviated fatty liver and inflammatory response in high-fat diet-fed mice [12]. Li et al. [13] proved that artemisinin had the regeneration of pancreatic beta-cell mass from alpha cells as a potential drug for the treatment of type 1 diabetes. However, in 1988 some people used* Artemisia* for clinical trials; the experiment found that it had hypoglycemic effect and a slight reduction in blood pressure, but there were only 15 cases in the group [14]. Then, what about the efficacy of artemisinin in type 2 diabetes?

In the present study, we have evaluated the effects of artemether on blood glucose, insulin resistance, islet and liver morphology, and beta-cell function in C57BL/KsJ db/db mice with spontaneous diabetes. The results show that artemether has beneficial effects on glucose homeostasis, insulin resistance, pancreas and liver architecture, the apoptosis of beta cells, and insulin secretion in db/db mice, which suggests that artemisinins may be useful in type 2 diabetes mellitus.

## 2. Materials and Methods

### 2.1. Reagents

Artemether (purity 98%) and methylcellulose were provided by DASF Bio-Tech Ltd (Nanjing, China). The samples were air-dried and then stored at 4°C. 1% methylcellulose was dissolved in distilled water, heating to 80°C with agitation as vehicle, and then stored at 4°C. Artemether suspension in 1% methylcellulose was prepared fresh daily before treatment.

### 2.2. Animals and Experimental Design

Ten male C57BL/KsJ-db/db mice and five C57BL/KsJ-db/+ mice (6–8 weeks of age) were procured from Better Biotechnology Co., Ltd. (Nanjing, China). Mice were housed in standard laboratory conditions (23 ± 1°C, 40–60% relative humidity, and a 12 hours light-dark cycle) in the experimental animal center of the First Affiliated Hospital, and College of Clinical Medicine of Henan University of Science and Technology. All mice were allowed free access to the normal chow diet and tap water. After two weeks of acclimatization, the mice were randomly divided into three groups (*n* = 5): normal control (NC, db/+, 1% methylcellulose, intragastric administration), diabetic control (DM, db/db, 1% methylcellulose, intragastric administration), and artemether treated (artemether, db/db, 200 mg/kg of artemether, intragastric administration). The treatment lasted for two weeks. At the end of the experiment, all animals were anesthetized with ether after fasting from eight o'clock to Sixteen o'clock. Whole blood was collected and serum was isolated for biochemical analysis. Then, a part of liver and pancreas were removed, rinsed, frozen in liquid nitrogen immediately, and stored at −80°C till analysis. Others were stored in formalin at room temperature. Food consumption and body weight were measured every three days.

### 2.3. Measurement of Blood Glucose, Serum Insulin, and Glucagon

Fasting blood glucose (FBG) was measured every three days via tail nick using a handheld glucometer (Roche ACCU-CHEK, Germany) after fasting from eight o'clock to Sixteen o'clock. Serum insulin level and glucagon level were determined by the RayBio mouse insulin ELISA and mouse glucagon EIISA.

### 2.4. Intraperitoneal Glucose and Insulin Tolerance Test

At the beginning and end of the experiment, the IPGTT and IPITT were performed over a one-day interval. After 12 hours of fasting (from twenty o'clock to eight o'clock), the animals received a 50% glucose solution (1.0 g/kg of body weight, China Otsuka Pharmaceutical Co., Ltd.) intraperitoneally. The plasma glucose concentration was evaluated in blood samples collected from the tails at 0, 15, 30, 60, 90, and 120 min after glucose injection. Briefly, IPITT was performed on the next day after 2 h of fasting. Insulin (0.5 IU/kg, Wanbang Biopharma) was injected into the peritoneal cavity. Glucose levels of the tail blood were determined at 0, 15, 30, 60, and 120 min after insulin injection.

### 2.5. Morphology

The pancreas and liver samples were fixed in 4% paraformaldehyde overnight and then processed for paraffin embedding, sectioning, and H&E staining. Microphotographs were captured with a light microscope.

### 2.6. Tunel Staining

The tissues of pancreas were embedded in paraffin. Sections (4 *μ*m) were cut as described previously. We operated according to the instruction of the TUNEL kit (Roche, Palo Alto, CA). And the sections were washed in PBS for 3 times before incubation in DAPI for 10 min and analyzing in a mounting medium under a fluorescence microscope. The number of positive cells in the 5 noncontinuous high power field of vision was observed and counted under the microscope.

### 2.7. Pancreas Immunofluorescence

Pancreas was harvested from male mice, postfixed in 4% paraformaldehyde, rinsed in 70% ethanol, and embedded in paraffin. Paraffin sections (4 *μ*m) were prepared. Pancreas sections were immunostained for insulin (guinea pig anti-insulin antibody, 1 : 800, Sigma, USA) and glucagon (mouse anti-glucagon antibody, 1 : 1000, Abcam, ab92517) and counterstained with DAPI (Olympus, Tokyo, Japan) to identify nuclei.

### 2.8. Statistical Analysis

All data were expressed as mean ± SEM, and the statistical significance was set to 5% (*P* < 0.05). The statistical analysis was performed with Graph Pad Prism software, and the differences were determined by Student's *t*-test.

## 3. Results

### 3.1. Effects of Artemether on Food Intake and Body Weight in db/db Mice

**Figure 1:**
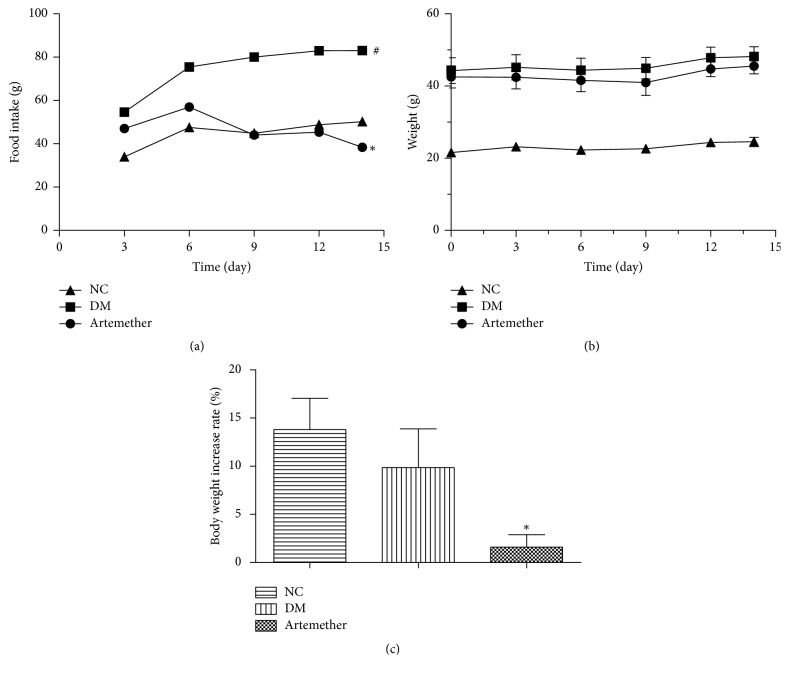
*Effects of artemether on food intake and body weight in db/db mice*. (a) Food intake. (b) Change in body weight. (c) The weight gain at the end of the experiment. Values are mean ± SEM for 4-5 mice. ^#^*P* < 0.05, versus NC group, and ^*∗*^*P* < 0.05, versus DM group.

As expected, db/db mice became obese by the tenth week. To examine the antiobesity effect of artemether on db/db mice, we measured food intake and body weight every 3 days. The intakes of DM group were increased compared to those of NC group (*P* < 0.05) (Figure 1(a)), whereas the intakes of artemether group were reduced slightly than the DM group (*P* < 0.05). However, as shown in Figure 1(b), the weights of three groups were slowly increased. The weights of artemether group were decreased. Though there were no significant differences among the groups, the body weight increase rates of artemether group were lower than NC group at the end of the experiment (*P* < 0.05) (Figure 1(c)).

### 3.2. Glucose Homeostasis Was Improved in db/db Mice Treated with Artemether

**Figure 2:**
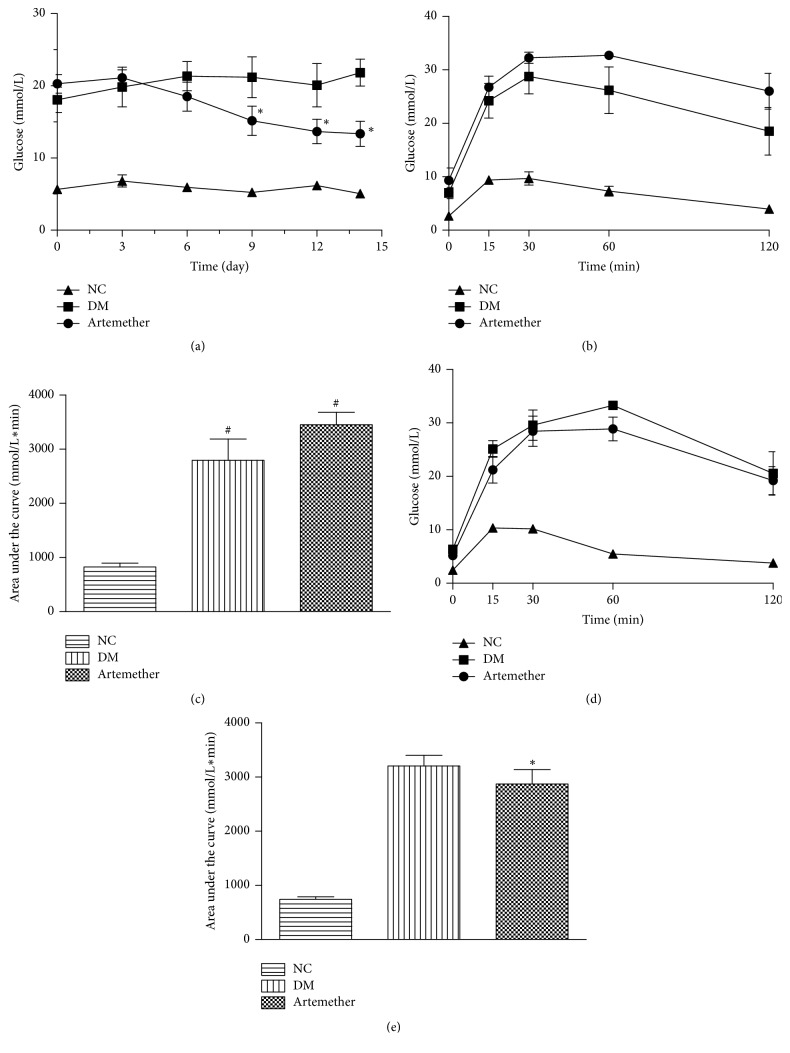
*Effects of artemether on glucose and glucose tolerance in db/db mice*. Ten-week-old mice were treated with 1% methylcellulose or with artemether (200 mg/kg) for two weeks. Intraperitoneal glucose tolerance tests (IPGTT) (1 g/kg) and fasting glucose were performed after overnight fasting. (a) shows the fasting blood glucose levels; *P* < 0.05, versus DM group. (b) shows intraperitoneal glucose tolerance test of mice at the start of experiment. Means and SEM are indicated for 4-5 animals from each group. (c) shows the area under the curve from the glucose tolerance test described in (b). (d) shows intraperitoneal glucose tolerance test of mice after the treatment of artemether. (e) shows the area under the curve from the glucose tolerance test described in (d). Measurements in individual animals are shown, and the means and SEM are indicated for the groups; ^#^*P* < 0.05, versus NC group, and ^*∗*^*P* < 0.05, versus artemether group in (c).

To determine the effect of artemether on blood glucose, we conducted an IPGTT at the start and end of the experiment and measured the fasting blood glucose every three days. As shown in Figure 2(a), the artemether group exhibited significantly reduced fasting blood glucose levels compared with the DM group after 9 days of intervention (*P* < 0.05). After administering a glucose solution, blood glucose levels in all groups peaked at 30 and 60 min, among which the db/db mice exhibited the higher blood glucose levels (Figure 2(c)). The AUCs were significantly higher in DM group than NC group (*P* < 0.05) (Figure 2(d)). Then after 2 weeks of artemether treatment, the blood glucose at each time point of the artemether group was decreased compared with the DM group though the AUCs of the db/db mice treated with artemether were of no significant difference compared with DM group. In fact the AUCs were decreased by 16.8% compared with that before intervening in artemether group (*P* < 0.05) (Figure 2(e)). This indicated that the treatment with artemether significantly improved glucose tolerance of db/db mice.

### 3.3. Artemether Improved Insulin Resistance in db/db Mice

**Figure 3:**
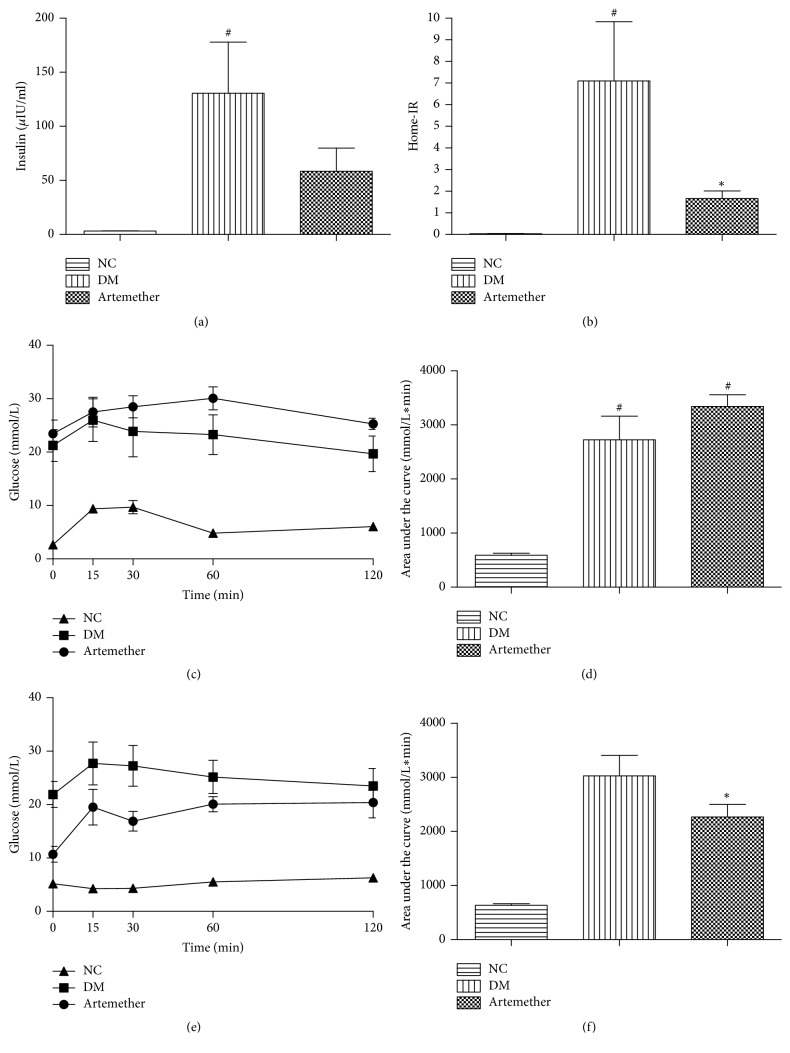
*Effects of artemether on insulin resistance in db/db mice*. At the beginning and the end of the treatment, intraperitoneal insulin tolerance tests (IPITT) (0.5 U/kg), and insulin levels were performed after 2 h of fasting. (a) Fasting insulin levels. (b) HOME-IR. (c) shows the result of IPITT of mice at the start of experiment. (d) shows the area under the curve from the insulin tolerance test described in (c). (e) shows IPITT of mice after the treatment of artemether. (f) shows the area under the curve from the insulin tolerance test described in (e). Values are mean and SEM for four-five mice. ^#^*P* < 0.05, versus NC group, and ^*∗*^*P* < 0.05, versus DM group in (d).

Does artemether affect insulin resistance in db/db mice? Fasting glucose and insulin levels were measured. As shown in the Figures 2(a) and 3(a), the fasting glucose and insulin levels of DM group were significantly increased than NC group (*P* < 0.05); however the results in mice treated with artemether were decreased. And we calculated HOMA-IR. The HOMA-IR of DM group was higher than NC group which suggested db/db mice had severe insulin resistance (Figure 3(b)). However we found the HOMA-IR of the Artemether group was decreased compared with the DM group (*P* < 0.05). We also performed an IPITT at the start and end of the study. As a result, the blood glucose at each time point was lower than that before intervention at the end of the experiment in artemether group (Figures 3(c) and 3(e)). And the AUCs of artemether group were significantly lower than those that before intervention (*P* < 0.05) (Figures 3(d) and 3(f)). Those results all proved that artemether improved insulin sensitivity and ameliorated insulin resistance of db/db mice.

### 3.4. Effects of Artemether on Islet Morphology Changes in db/db Mice

**Figure 4:**
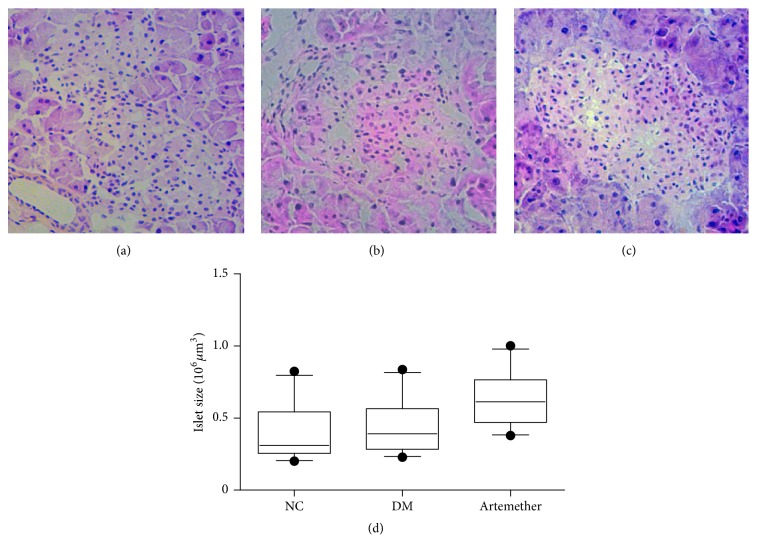
*Effects of artemether on islet morphology changes in db/db mice*. (a) NC group. (b) DM group. (c) Artemether group (200 mg/kg for two weeks). Scale bar, 50 *μ*m. (*n* = 5). (d) Quantification of islets size in sections from mice pancreas. Boxes and whiskers are shown, 10%–90% confidence intervals.

As shown in Figure 4(a), light microscope examination of HE stained sections revealed the appearance of pancreatic acinar structures in NC group. Pancreatic islets of normal pancreatic tissue were round or oval and had clear boundary, with the cells arranged in neat, medium size, uniform distribution. The acini were well arranged and organized in an orderly fashion. In the DM group, pancreatic morphologies were abnormal and the boundaries were not clear, the cell number decreased, and vacuolar degeneration existed (Figure 4(b)). Then Figure 4(c) showed that the islet morphologies of artemether group were better than those of DM group, and the boundaries were clear and the cell numbers also were increased. And total islet sizes of db/db mice treated with artemether were increased compared with DM group (Figure 4(d)).

### 3.5. Effects of Artemether on Hepatic Steatosis in db/db Mice

**Figure 5:**
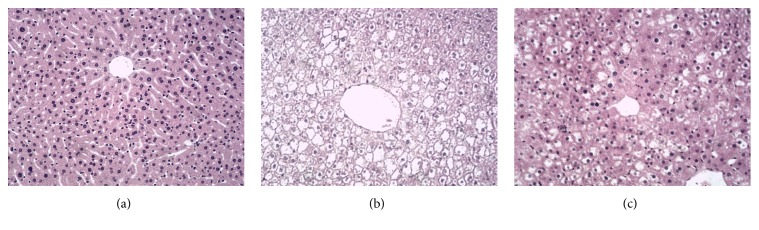
*Effects of artemether on hepatic steatosis in db/db mice*. (a) NC group. (b) DM group. (c) Artemether group (200 mg/kg for two weeks). Scale bar, 100 *μ*m. (*n* = 5).

In order to evaluate the effect of artemether on fatty liver in obese mice, we performed liver HE staining. In light microscopy, as shown in Figure 5(a), the liver cells of NC group were arranged neatly and there were almost no lipid droplet vacuoles; there were a lot of lipid droplets in the liver cells of DM group, and the volume of liver cells increased and the shape of liver cells changed (Figure 5(b)); then we can see that there were hepatic steatosis in artemether group which were reduced significantly compared to those of DM group, but liver cells still were arranged in disorder. Therefore, we may conclude that artemether can ameliorate fatty liver.

### 3.6. Artemether Reversed the Damage of Pancreatic Beta-Cell in db/db Mice

**Figure 6:**
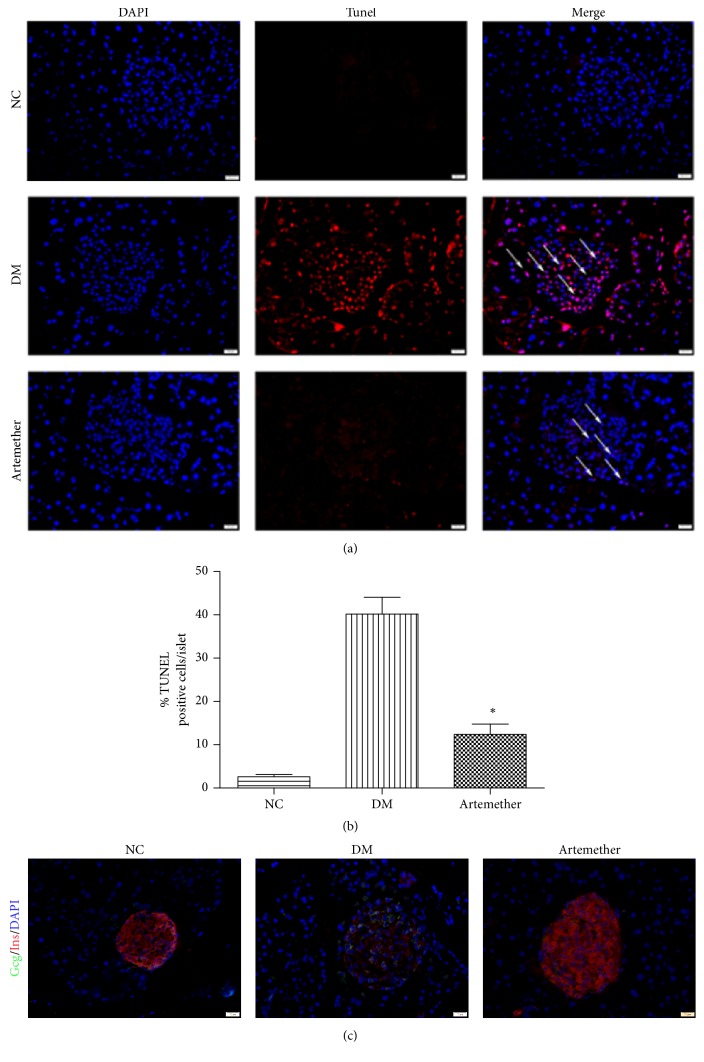
*Effect of artemether on the damage of pancreatic beta-cell in db/db mice*. (a) Representative images (×40 magnification) of DAPI and TUNEL islets staining. Scale bar, 20 *μ*m. (b) Quantification of apoptosis rate of islet cells in db/db mice. ^*∗*^*P* < 0.05, versus DM group. *n* = 5. (c) Representative staining for insulin, glucagon, and DAPI in mice pancreas. Scale bar, 20 *μ*m. The arrows refer to the apoptotic cells.

To evaluate the effect of artemether on pancreatic beta-cell in db/db mice, we performed the TUNEL assay to evaluate beta-cell apoptosis in islets. We found that treatment with artemether resulted in a significant decrease of beta-cell apoptosis in islets of db/db mice (*P* < 0.05) (Figures 6(a) and 6(b)). We also observed the effect on insulin and glucagon secretion. Artemether significantly improved insulin secretion in islets, relative to diabetic controls (Figure 6(c)). These results suggested that artemether could protect beta-cell in db/db mice.

## 4. Discussion

The present study is the first study to prove the antidiabetic and antiobesity effects of artemether in type 2 diabetic db/db mice. In this model, the leptin receptor genetic mutation occurred spontaneously. As a consequence of a loss of leptin function, the db/db mice show many of the characteristics of type 2 diabetic patients, including obesity, hyperglycaemia, and insulin resistance [15]. Excitingly, we found that artemether could prevent obesity, reduce blood glucose level, improve insulin resistance, ameliorate fatty liver, and protect pancreatic beta cells in our study.

Our results clearly demonstrate that artemether significantly improved glucose homeostasis in this animal model. The blood glucose levels began to decrease after 9 days of treatment, and the AUCs of IPGTT in the artemether group were reduced by 16.8% at the end of experiment. The result of the IPGTT indicates that artemether increased the amount of peripheral glucose uptake and reduced postprandial blood glucose levels, which have been reported to be an independent risk factor for the development of macrovascular complications [16]. Moreover, the PPAR*γ* agonists have been widely used in type 2 diabetes mellitus, such as thiazolidinone drugs (TZDs). Although these drugs can increase insulin sensitivity and decrease blood glucose, they also promote lipid storage and fat formation, causing side effect of increased body weight [17]. Surprisingly, we firstly found that artemether not only increased insulin sensitivity and improved insulin resistance, but also reduced food intake and body weight increase rate, which makes up for the shortcomings of this type of insulin sensitizer. In past studies, artemisinins have been shown to prevent obesity and inhibit adipogenesis [18–20]. But in our experiments artemether did not reduce the body weight of db/db mice significantly; possibly it was related to the length of treatment. We also found that artemether had a therapeutic effect on fatty liver in db/db mice. However, Kim et al. also found that in high-fat diet-fed mice* Artemisia annua* leaf extract may prevent the development of hepatic fibrosis and reduce lipid accumulation and inflammation in the liver [12]. And artemisinins have anti-inflammatory and antioxidant activities [21], which may be involved in the treatment of diabetes. They all indicate that artemisinin may be beneficial for the prevention of obesity-induced metabolic syndromes, promising to treat type 2 diabetes. We also confirmed that artemether protected beta cells function by reducing the apoptosis of pancreatic beta cells and increasing insulin secretion in db/db mice. In cell experiments, artesunate was proved to protect pancreatic beta cells against cytokine-induced damage via SIRT1 inhibiting NF-*κ*B activation [22]. And in type 1 diabetic rat artemisinins as approved drugs were identified that can confer *β* cell characteristics to *α* cell. [14]. But some researchers have questioned this mechanism recently. They thought artemether did not induce the transdifferentiation of alpha cells and suppressed glucose uptake and prevents insulin secretion [23]. So the hypoglycemic mechanism of artemether remains to be further confirmed.

In conclusion, we demonstrated that artemether significantly improved glucose homeostasis and insulin resistance, had the potential activity to prevent obesity, reduced the severity of fatty liver, and protected pancreatic beta cells in db/db mice. Hence, artemether might be an effective drug for type 2 diabetes. Studies in our laboratory are in progress to investigate the antidiabetic and antiobesity mechanism and observe if there are additive or synergistic effects. We will perform a small sample of clinical trials to identify the effects. We believe that artemisinin and its derivatives would benefit more humans in the future.
